# Internet-based cognitive behavioral therapy for sexual dysfunctions in women treated for breast cancer: design of a multicenter, randomized controlled trial

**DOI:** 10.1186/s12885-015-1320-z

**Published:** 2015-04-28

**Authors:** Susanna B Hummel, Jacques JDM van Lankveld, Hester SA Oldenburg, Daniela EE Hahn, Eva Broomans, Neil K Aaronson

**Affiliations:** 1 Division of Psychosocial Research and Epidemiology, The Netherlands Cancer Institute, Plesmanlaan 121, 1066 CX, Amsterdam, The Netherlands; 2 Faculty of Psychology and Educational Sciences, Open University, Valkenburgerweg 177, 6419 AT, Heerlen, The Netherlands; 3 Department of Surgical Oncology, The Netherlands Cancer Institute, Plesmanlaan 121, 1066 CX, Amsterdam, The Netherlands; 4 Department of Psychosocial Counseling, The Netherlands Cancer Institute, Plesmanlaan 121, 1066 CX, Amsterdam, The Netherlands; 5 Department of Adult Care, Virenze Institute of Mental Health Care, ‘t Goylaan 7, 3525 AA, Utrecht, The Netherlands

**Keywords:** Breast cancer, Sexual dysfunction, Intimacy, Cognitive behavioral therapy, Internet-based, Randomized Controlled Trial

## Abstract

**Background:**

Sexual dysfunction is a prevalent, long-term complication of breast cancer and its treatment and can be treated effectively with face-to-face sexual counselling. However, relatively few women actually opt for face-to-face sex therapy, with many women indicating that it is too confronting. Internet-based interventions might be a less threatening and more acceptable approach, because of the convenience, accessibility and privacy it provides. Recent studies have demonstrated the efficacy of internet-based programs for improving sexual functioning in the general population. The objective of the current study is to investigate the efficacy of an internet-based cognitive behavioral therapy (CBT) program in alleviating problems with sexuality and intimacy in women who have been treated for breast cancer.

**Methods/design:**

In a multicenter, randomized controlled trial we are evaluating the efficacy of an internet-based CBT program in reducing problems with sexuality and intimacy in breast cancer survivors. Secondary outcomes include body image, marital functioning, psychological distress, menopausal symptoms, and health-related quality of life. We will recruit 160 breast cancer survivors (aged 18-65 years) with a formal DSM-IV diagnosis of sexual dysfunction from general and academic hospitals in the Netherlands. Women are randomized to either an intervention or waiting-list control group. Self-report questionnaires are completed by the intervention group at baseline (T0), ten weeks after start of therapy (T1), post-treatment (T2), 3 months post-treatment (T3), and 9 months post-treatment (T4). The control group completes questionnaires at T0, T1 and T2.

**Discussion:**

There is a need for accessible and effective interventions for the treatment of sexual dysfunctions in breast cancer survivors. This study will provide evidence about the efficacy of an internet-based approach to delivering a CBT intervention targeted specifically at these sexual health issues. If proven to be effective, internet-based CBT for problems with sexuality and intimacy will be a welcome addition to the care offered to breast cancer survivors. Hopefully this therapy will lower the barrier to seeking help for these problems, resulting in improved quality of life after breast cancer.

**Trial registration:**

The study is registered at ClinicalTrials.gov (NCT02091765).

## Background

Breast cancer is the most common type of cancer among women in the Netherlands [1]. Improved breast cancer screening and treatment have resulted in increased survival rates [2]. Consequently, more interest and research has focused on the health-related quality of life (HRQL) of breast cancer survivors, including issues of sexuality and intimacy.

The prevalence rates for sexual dysfunctions as a result of breast cancer treatment vary between 30% and 100% [3-7]. Breast cancer survivors (BCS) experience worse sexual functioning compared to women without a history of cancer [8-10]. Frequently reported problems include decreased sexual desire (23-64%), decreased sexual arousal or vaginal lubrication (20-48%), anorgasmia (16-36%) and dyspareunia (35-38%) [3].

The different components of breast cancer treatment can all directly or indirectly affect sexual functioning [3]. Previous studies have shown that women who have received chemotherapy are at a higher risk of developing sexual dysfunctions than women who have not undergone this treatment [8,11-17], regardless of the type of surgery [14,18]. Chemotherapy can cause premature, abrupt menopause, leading to reduced sexual desire in some women [19]. It can also induce vaginal dryness and atrophy, which subsequently can affect sexual functioning [6,8,12,13,15,20]. Results with regard to endocrine treatment are somewhat mixed, but studies show that tamoxifen and aromatase inhibitors can lead to sexual problems [21-27]. The evidence pertaining to the effect of surgery on sexual functioning is mixed [28-30], with some studies showing that women who undergo a mastectomy report more problems in sexual functioning than women who receive breast conserving therapy [28,31,32], while other studies have not found an association between type of surgery and sexual functioning [18,29]. More consistent is the finding that mastectomy more often results in compromised body image than does breast conserving treatment [4,13,28]. Other common complaints after breast cancer treatment are concerns about sexual attractiveness and femininity, fatigue, anxiety and depression, fear of loss of fertility, and overall decreased HRQL [3,18,33,34]. Emotional well-being and the quality of the partner-relationship can also be affected by the distress surrounding diagnosis and treatment [35-37]. Although the diagnosis and treatment of any type of cancer can cause problems in sexual functioning [3], breast cancer raises particular concerns because of the importance of the breast in feminine sexuality and the breast as a source of erotic pleasure and stimulation [33].

Sexual dysfunctions can be treated effectively with face-to-face forms of sex therapy [38-41]. Sex therapy typically comprises a flexible treatment program including a number of elements that can be tailored to the needs of individuals and couples. It typically involves behavioral components derived from the sex therapy developed by Masters and Johnson [42], i.e. psycho-education about sexuality and sexual dysfunction, a temporary ban on intercourse, and sensate focus exercises. A ban on intercourse can break the vicious cycle of fear of sexual intercourse and subsequent negative experience and disappointment, and offers the opportunity for positive experiences by eliminating or reducing performance demand [43]. Sensate focus exercises form a hierarchically structured exercise program, through which partners gradually reintroduce the consecutive phases of sexual contact. The exercises are targeted at becoming more comfortable with one’s own body and achieving sexual intimacy with one’s partner, both physically and emotionally. Other goals are to discover new approaches to sexual stimulation, and to encourage communication between partners about sexual experiences, sexual desires and sexual boundaries. These behavioral elements of sex therapy are usually combined with cognitive therapy [40,43]. Through cognitive therapy, therapist and client aim to detect and modify the client’s dysfunctional, disturbing cognitions regarding sexuality that arise during exercises. Via the method of cognitive restructuring, the dysfunctional cognitions are replaced by more functional appraisals. Sex therapy is often delivered in a couple format, but individual applications and group therapy formats are also described in the literature [44,45].

The efficacy of different types of face-to-face therapy for female sexual dysfunction (FSD) has been demonstrated, including sexual desire and sexual arousal disorder [40,46,47], orgasmic disorder [48,49], sexual pain [50,51], and vaginismus [52,53]. Several modified treatment programs have been developed and evaluated for breast cancer survivors [44,54]. Interventions with stronger effects tend to be couple-focused and include treatment components that educate both partners about the woman’s diagnosis and treatment, promote couples’ mutual coping and support processes, and include treatment components that make use of specific sex therapy techniques addressing sexual and body image concerns [44,54].

Despite the availability of effective treatments for sexual dysfunctions, there is a significant discrepancy between the self-reported need for professional sexual health care in cancer survivors and the actual uptake of care [5,55]. Kedde et al. [5] reported that only 40% of BCS who felt a need for care actually consulted a health professional. Hill et al. [55] reported that, although over 40% of gynaecologic cancer and breast cancer survivors expressed interest in receiving professional care, only 7% had ever actually sought such care.

Although sexual functioning is an important issue, health care professionals may be reluctant to query breast cancer patients about sexual problems during medical consultations, due to time constraints, embarrassment, lack of knowledge and experience in this area, and/or lack of resources to provide support if needed [56,57]. It may also be difficult for patients to initiate discussion about their sexual difficulties with their health care professional [58-60]. It has been suggested that when reporting sensitive or potentially stigmatizing information, individuals may feel more comfortable undergoing assessment and treatment via the internet [61,62]. This idea is supported by a survey [de Blok G. Thesis on the outpatient clinic for sexuality and breast cancer of The Netherlands Cancer Institute. Unpublished manuscript] that was conducted in women who attended an informational meeting of a sexuality and breast cancer clinic, but who subsequently did not follow-up for an appointment for face-to-face counselling. While some women indicated that they did not consider treatment of their sexual problems to be necessary, others indicated that they did not wish to undergo such treatment in a hospital-setting, or that the face-to-face setting of the counselling formed too great a barrier. Many respondents suggested that internet-based therapy would be a less threatening and more acceptable approach. The advantages of internet-based therapy include privacy, convenience and accessibility [63-65], all of which may be particularly attractive in the area of sexual problems.

There is growing evidence that internet-based CBT is an effective method to treat a range of psychosocial problems [66-73]. More recently, internet-based CBT programs for sexual dysfunctions have been developed and tested [45,65,74-77]. However, most of these online interventions have focused on male sexual dysfunctions [74,75,77-80]. Early trials have demonstrated the applicability and effectiveness of online CBT for FSD in the general population [76], and of an online intervention for sexual problems in breast cancer survivors [81]. However, the efficacy of an internet-based CBT for sexual problems in BCS has not yet been researched.

In this article, we describe the design of a randomized, controlled, multicenter trial that evaluates the efficacy of an internet-based CBT program for sexual dysfunctions in women who have been treated for breast cancer. We hypothesize that women in the internet-based CBT group will report a significantly greater improvement in sexual functioning and intimacy than women in a waiting-list control group. Secondarily, we hypothesize that women who undergo the internet-based CBT will report significantly less psychological distress and fewer menopausal symptoms, and a significantly greater improvement in body image, marital functioning and HRQL than women in the control group.

## Methods

**Figure 1:**
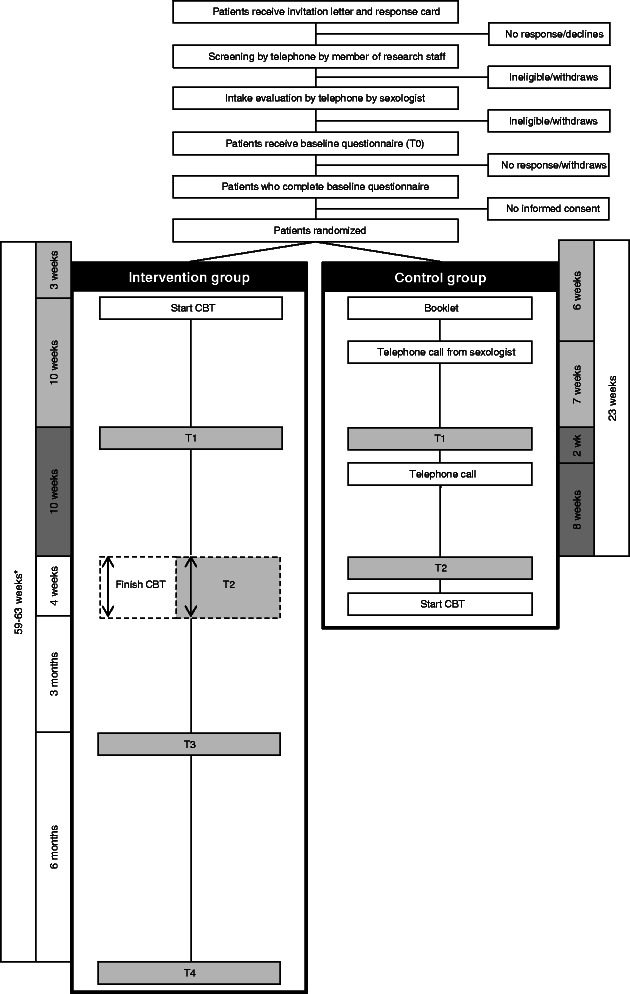
Overview of study procedures. *The total duration of study participation is dependent on the duration of the CBT.

In this study, patients are randomized to either an intervention group or a waiting-list control group. Women in the intervention group will undergo an internet-based CBT aimed at alleviating problems with sexuality and intimacy. The design of the trial and the anticipated flow of participants are displayed in Figure 1. The trial has been approved by the Institutional Review Board of The Netherlands Cancer Institute (under number NL44153.031.13), as well as by all review boards of the hospitals from which patients are being recruited (for a list of the participating hospitals, see the Acknowledgements section). Patient recruitment and data collection started in September, 2013.

### Study sample

The study sample will be composed of 160 women fulfilling the following inclusion criteria: (1) age 18-65 years (the upper limit of 65 years is not based on any assumption regarding the salience of sexuality with increasing age, but on the smaller chance of access to internet in this age group); (2) a history of histologically confirmed breast cancer (stages: T1-T4, N0-N1 and M0); (3) a diagnosis of breast cancer six months to five years prior to study entry; (4) completion of breast cancer treatment (with the exception of endocrine therapy and immunotherapy); (5) disease-free at time of study entry; (6) a basic fluency in the Dutch language (for assessment and therapy purposes); and (7) a formal diagnosis of sexual dysfunction according to the criteria of the Diagnostic and Statistical Manual of Mental Disorders-IV [82] (to be established by an experienced sexologist during an intake interview). Single as well as partnered women can participate in the study. Sexual orientation is irrelevant for eligibility.

Exclusion criteria are: (1) no access to internet; (2) serious cognitive or psychiatric problems (i.e., depression, alcohol dependency, or psychotic disorders) as determined on the basis of the Mini International Neuropsychiatric Interview [83]; (3) treatment for another type of cancer (with the exception of cervix carcinoma in situ and basal cell carcinoma); (4) presence of severe relationship problems for which the internet-based program is not appropriate; (5) participation in a concurrent therapy program to alleviate problems with sexuality or intimacy; (6) participation in a concurrent CBT program for other psychological problems; and (7) participation in another trial investigating problems with sexuality/intimacy.

### Recruitment and randomization

Patients are recruited from 10 community and university hospitals in the Netherlands and are identified through the hospital registries by their physician, or by means of the database of the Netherlands Cancer Registry. Selected patients are sent an invitation letter describing the study and internet-based therapy, and are asked to return a response card to indicate if they are interested in participation. In case of no interest, women are asked to specify their reason(s) for this on the card. In the absence of a response, a reminder is sent three weeks after the first mailing. Women who are not interested in participation or who do not respond to the reminder are not contacted again.

Interested women are screened for eligibility twice: first by a member of the study staff and subsequently by a sexologist. In these interviews, more information about the study procedures and therapy is given and eligibility criteria are checked. The sexologist carries out a diagnostic interview to determine final eligibility of the woman, and the Mini International Neuropsychiatric Interview [83] is completed. All sexologists involved in the study are female, and have undergone special training in the application of the internet-based CBT program.

Eligible women are sent a baseline questionnaire (T0) and an informed consent form. Study questionnaires can be completed online or in a paper-and-pencil format. The baseline questionnaire assesses sociodemographic and medical background variables, and the study outcomes. If the score of the marital adjustment subscale of the Maudsley Marital Questionnaire [84] (see ‘Study measures’ section) exceeds the cut-off score of 35, the eligibility of the participant is discussed once more with the sexologists to determine if the nature and severity of the relationship problems would recommend treating these problems prior to tackling the sexual dysfunction, thus resulting in exclusion from study participation.

Consenting women are randomized to either an intervention group (n = 80) or a waiting-list control group (n = 80) using the minimization technique, with type of surgery (breast conserving therapy; mastectomy only; mastectomy with breast reconstructive surgery), current endocrine treatment for breast cancer (yes; no), time since breast cancer diagnosis (<1 year; 1-3 yrs; 3-5 yrs), and menopausal status (premenopausal; postmenopausal) as stratification variables.

### Study arms

#### Intervention group: internet-based CBT program

Each woman is assigned a sexologist who guides her through the internet-based CBT program. Contact with the therapist is web-based, via a secured, password-protected website. From a total of 10 modules, the sexologist selects four to five modules that best fit the sexual problems of the client. The internet-based CBT program was originally developed for use in the general population at Virenze, a mental health center located in Utrecht, the Netherlands. The program was adapted for use specifically for breast cancer survivors. This involved editing and adding text related to the physical and psychosexual problems often experienced by women who have had breast cancer. The therapists are licensed psychologists and sexologists who have undergone additional training in issues relating to breast cancer, and in the use of the internet program.

A description of the content of the different program modules is provided in Table 1. Each module contains several interventions, each of which comprises the following elements: (1) introduction, (2) psycho-education, (3) “homework” assignments (e.g., registration exercises; discuss intimacy with partner; sensate focus exercises) and (4) reporting back to the therapist and receiving feedback on the homework assignments. The modules can be used in varying order, leading to a tailored and flexible treatment program consisting of a maximum of 20 therapy sessions that are completed within a period of 24 weeks. A minimum of five sessions is considered the lower limit of sessions required in order to expect an effect. The average time investment for a participant is 90-120 minutes per week. Women are motivated by the sexologist to involve their partner in the treatment, but partner involvement is not mandatory. The time limit within which the therapist should respond to incoming messages from a client is set at five working days. Weekly contact between therapist and client is pursued. After 10 weeks of treatment, a mid-term evaluation is held to reflect on the progress so far, and to adjust goals, if necessary, for the remaining part of treatment. The internet-based CBT program is provided at no cost to the woman.

**Table 1:** **Description of therapy modules**

**Module 1: Put your problem into words**	In this module the client describes her sexual problems, and learns how sexuality can be influenced by the treatment of breast cancer. The sexual response curve and female sexual dysfunctions are elaborated on. Furthermore, information is given about what intimacy is and how it interplays with sexuality. Women are encouraged to discuss their sexual problems with their partner.
**Module 2: How is my relationship doing?**	In this module the client explores the level of intimacy in her relationship, becomes aware of the amount of quality time spent with the partner, and receives psycho-education about sex and intimacy. The importance of open communication with the partner is discussed, and advice is given on how to improve communication with regard to intimacy and in particular sex. The couple evaluates how their relationship and sex life has been influenced by the diagnosis and treatment of breast cancer.
**Module 3: Sex and my body**	In this module sensate focus therapy is introduced. The first steps of the hierarchically structured exercise program are completed. An introduction is given with regard to the influence of thoughts and external stimuli on the experience of sex. Attention is also paid to possible tension in the pelvic floor and methods to relax this part of the body.
**Module 4: Focus my attention**	In this module the client receives task concentration training in order to learn to focus her attention on sexual experiences in such a way that it is beneficial to the client.
**Module 5: Explore my body**	In this module sensate focus therapy is elaborated on and the hierarchically structured exercise program is completed. The client reports on her experiences with the homework exercise within a cognitive behavioral framework.
**Module 6: Discovering my sexual arousal feelings (version for male partners)**	The topics of this module are similar to the female version (see module 7), but are written from a male perspective.
**Module 7: Discovering my sexual arousal feelings (female version)**	In this module psycho-education is provided about the female body and genitals, female sexual dysfunction, genital stimulation, sexual techniques, and the male body and genitals. Accompanying exercises are provided for each subject, including, for example, exposure exercises for sexual pain disorders. Attention is also paid to the importance of and ways to discuss sexual feelings and preferences with the partner.
**Module 8: Change my thoughts**	In this module the influence of thoughts on feelings and behavior is explained, and the client’s dysfunctional cognitions with regard to sex and intimacy are identified. Via the method of cognitive restructuring these cognitions are replaced by more functional, adaptive thoughts.
**Module 9: My sexual preferences**	In this module the client’s sexual development, sexual needs, myths and beliefs about sex are evaluated. The client is encouraged to talk about her sexual preferences with her partner, and an action plan for behavior change is created.
**Module 10: Relapse prevention**	In this module the client reflects on her former automatic behavior and possible risk factors for relapse. A plan of action is generated to use in the event of a relapse.

#### Waiting-list control group

Participants in the control group are asked to refrain from undergoing any psychological or medical interventions for sexual problems during their participation in the study. To increase the likelihood that women will remain in the study until they are offered the CBT program, they are provided with a booklet addressing questions about sexuality and cancer. Additionally, six weeks after randomization and upon completion of the second questionnaire, women in the control group are contacted by telephone to address any questions or comments that they may have about their participation in the study, and to reconfirm that they will be eligible for the internet-based CBT program upon their completion of the study.

### Data collection

#### Patients

Patients in both study arms complete a battery of self-report questionnaires at equivalent moments in time for the first three assessments (T0: baseline; T1: 10 weeks after start of therapy (intervention group) or 13 weeks after randomization (control group); and T2: post-treatment (intervention group) or 23 weeks after randomization (control group), see Figure 1). To achieve an equivalent average assessment time for both groups, women in the intervention group complete T2 post-treatment, but always between 20 and 24 weeks after start of therapy. Women who finish the CBT prior to 20 weeks complete T2 20 weeks after start of therapy. Women in the intervention group also complete questionnaires three months post-treatment (T3) and nine months post-treatment (T4). The control group is not asked to complete T3 or T4, but rather is given the opportunity to undergo the intervention following completion of T2. This was done because it was not deemed ethically acceptable to withhold the intervention from women in the control group for the prolonged period of time that would be required if they were to complete all follow-up questionnaires (i.e., approximately one year after study enrollment). To minimize respondent burden, the T4 questionnaire only includes the questionnaires assessing the primary outcome measures. In every questionnaire, women in both the intervention group and control group are asked if they pursued any other activities to reduce their sexual problems (e.g., use of vaginal lubricant, relaxation exercises). A reminder is sent to participants who do not complete and return the questionnaire within one week. If a woman does not complete the questionnaire in the week after the reminder, she is contacted by telephone.

#### Partners

Only partners of the women in the intervention group are asked to complete questionnaires regarding problems with sexuality and intimacy (male partner: IIEF [85], female partner: FSFI [86]), and relational functioning (PAIR Inventory [87], MMQ [84]) at the same points in time as the participants.

### Study measures

#### Sociodemographic and clinical data

Sociodemographic data and clinical data are obtained during the screening interview and via the baseline questionnaire. Sociodemographic data include age, education, relational status, living situation and work status. Clinical data are collected from the medical records and via self-report, and include date of breast cancer diagnosis, treatment (type of surgery, chemotherapy, radiotherapy, endocrine therapy, immunotherapy), medication use and comorbidity.

#### Outcome measures

Detailed descriptions of the outcome measures are provided in Table 2. Briefly, the primary outcome measures include standardized self-report questionnaires assessing problems with sexuality and intimacy. These include the Sexual Activity Questionnaire [88,89], the Female Sexual Function Index [86,90], the Female Sexual Distress Scale-Revised [91] and the PAIR Inventory [87]. Secondary outcome measures include standardized self-report questionnaires assessing body image (EORTC QLQ-BR 23 Body image subscale [92]), menopausal symptoms (FACT-ES ESS-18 [93]), marital functioning (MMQ [84]), psychological distress (HADS [94,95]) and HRQL (SF-36 [96,97]). The International Index of Erectile Function [85] is used to assess sexual functioning in male partners.

**Table 2:** **Study outcome measures and corresponding questionnaires**

Variable	Questionnaire	Details
**Primary outcomes**		
Sexual functioning	SAQ [88,89]	• Assesses sexual functioning
		• 10 items; 4-point Likert scales
		• Subscales: pleasure; discomfort; habit
		• Subscale scores: pleasure 0-18; discomfort 0-6; habit 0-3; higher score indicates higher levels of pleasure; lower score indicates lower levels of discomfort; habit is a single item (0 ‘less sexual activity than usual’ to 3 ‘much more sexual activity than usual’)
		• Time frame: past month
		• Test retest kappa: 0.50-0.76
	FSFI [86,90]	• Assesses sexual functioning
		• 19 items; 5- and 6-point Likert scales
		• Subscales: desire; arousal; lubrication; orgasm; satisfaction; pain
		• Total score*: 2-36/Subscale scores*: desire 1.2-6; arousal 0-6; lubrication 0-6; orgasm 0-6; satisfaction 0.8-6; pain 0-6; higher score indicates better sexual functioning
		• Time frame: past 4 weeks
		• Cronbach’s alpha: >0.82
	FSDS-R [91,99]	• Assesses distress related to sexual dysfunction
		• 13 items; 5-point Likert scale (0 ‘never’ to 4 ‘always’)
		• Total score: 0-52; higher score indicates higher level of sexual distress
		• Time frame: past 30 days
		• Cronbach’s alpha: >0.88
Intimacy	PAIR Inventory [87]	• 36 items; 5-point Likert scale (0 ‘strongly disagree’ to 4 ‘strongly agree’)
		• Subscales: emotional intimacy; social intimacy; sexual intimacy; intellectual intimacy; recreational intimacy; conventionality
		• Subscale score*: 0-96; higher score indicates higher levels of intimacy
		• Time frame: ‘how the relationship is now’
		• Cronbach’s alpha: 0.70-0.80
**Secondary outcomes**		
Body image	QLQ-BR23 Body Image subscale [92]	• 4 items; 4-point Likert scale (1 ‘not at all’ to 4 ‘very much’)
		• Score: 0-100; higher score indicates higher level of functioning
		• Time frame: past week
		• Cronbach’s alpha: 0.69-0.91
Menopausal symptoms	FACT-ES ESS-18 [93]	• 18 items; 5-point Likert scale (0 ‘not at all’ to 4 ‘very much’)
		• Score range: 0-72; higher score indicates fewer menopausal symptoms
		• Time frame: past 7 days
		• Cronbach’s alpha = 0.79
Marital functioning	MMQ [84]	• 20 items; 9-point Likert scale (range 0-8)
		• Scales: marital adjustment (M); sexual adjustment (S); general life adjustment (GL)
		• Scale scores*: S + GL: 0-40; M: 0-80; higher score indicates greater dissatisfaction in the specific domain
		• Time frame: past 2 weeks
		• Cronbach’s alpha in normal vs. distressed group: M = 0.88/0.87; S = 0.64/0.82; GL = 0.60/0.68
Psychological distress	HADS [94,95]	• 14 items; 4-point Likert scale (range 0-3)
		• Subscales: depression (HADS-D); anxiety (HADS-A)
		• Total score: 0-42/Subscale scores: 0-21; higher score indicates more psychological distress
		• Time frame: past week
		• Cronbach’s alpha: HADS-A: 0.68-0.93; HADS-D: 0.67-0.90
Health-related quality of life	SF-36 [96,97]	• 36 items; dichotomous and 3- to 6-point Likert scales
		• Subscales: physical functioning; role limitations due to physical health problems; bodily pain; social functioning; general mental health; role limitations due to emotional problems; vitality; general health perceptions
		• Subscale score*: 0-100; higher score indicates higher levels of functioning/well-being
		• Time frame: past week
		• Cronbach’s alpha = 0.66-0.93 (mean: 0.84)
Sexual functioning (male partners)	IIEF [85]	• 15 items; 5-/6-point Likert scale (0-5 or 1-5)
		• Subscales: erectile function (EF); orgasmic function (OF); sexual desire (SD); intercourse satisfaction (IS); overall satisfaction (OS)
		• Total score: 5-75/Subscale scores: EF 1-30; OF 0-10; SD 2-10; IS 0-15; OS 2-10; higher score indicates a higher level of functioning in specific domain
		• Time frame: past 4 weeks
		• Cronbach’s alpha: 0.73-0.99

*The score is calculated based on weighted items.

FACT-ES ESS-18 = Functional Assessment of Cancer Treatment-Endocrine Symptoms, Endocrine Symptom Subscale; FSDS = Female Sexual Distress Scale; FSFI = Female Sexual Function Index; HADS = Hospital Anxiety and Depression Scale; IIEF = International Index of Erectile Function; MMQ = Maudsley Marital Questionnaire; PAIR Inventory = Personal Assessment of Intimacy in Relationships Inventory; QLQ-BR23 = EORTC breast cancer-specific quality of life questionnaire; SAQ = Sexual Activity Questionnaire; SF-36 = 36-Item Short Form Health Survey.

### Compliance with the internet-based CBT program

The level of compliance is established via a question that is posed to the sexologist at the completion of therapy: ‘How many of the total number of therapy sessions that you considered to be optimal for this client has the client actually completed?’. This question is answered on a five-point scale, ranging from ‘the client has done all of the sessions I deemed necessary (100%)’ to ‘the client has not/barely done the sessions I deemed necessary (less than 25%)’. Additionally, the actual number of completed modules and interventions are extracted from the client’s records at the mental health center where the internet program is housed. Additionally, at completion of therapy, both the client and the sexologist are asked to indicate the frequency with which homework assignments were completed on a five-point scale (always-frequently-occasionally-rarely-never), and to indicate the reasons for not completing all homework assignments, if applicable. Women who do not complete the internet-based CBT program are asked to indicate their reason(s) for discontinuation (e.g., therapy was too intensive, online therapy was not suitable, illness). Every effort will be made to obtain all questionnaires of all participants, regardless of whether they do or do not complete their therapy.

### Patients’ evaluation of the intervention program

Upon completion of the CBT, women in the intervention group are asked to complete an evaluation questionnaire about the program. Questions are posed about their satisfaction with the program, the perceived efficacy of the program in alleviating sexual problems, their satisfaction with the choice of modules and exercises, the usability of the program, if they would recommend the treatment to other women experiencing similar problems, and if they would suggest any changes to the program. Women who discontinue the CBT program are asked the same questions.

A subset of women (approximately 15 from the intervention group) will be asked to participate in an evaluation interview by telephone. This semi-structured interview covers the same topic areas as addressed by the self-report evaluation questionnaire, allowing women to provide feedback in a more narrative form. Where applicable, women will also be asked if their partner would be willing to share his or her experience with the program.

### Statistical issues

#### Power calculation

The SAQ, FSFI, FSDS-R and PAIR Inventory are the primary outcome measures on which sample size calculations are based. With a total sample of 130 women (65 per group), and under the assumption of no interaction, the study will have a 80% power to detect a 0.5 standard deviation difference (Cohen’s effect size [98]) for the main effects of the internet-based CBT program, with the p-value set at 0.05 (two-sided test). A 0.5 standard deviation difference is considered to be indicative of clinically meaningful differences in self-reported symptom experience [98].

We will recruit 160 women into the study, to allow for an attrition rate of approximately 20% (i.e., women who discontinue participation in the study entirely, including failure to complete all follow-up questionnaires). Those women who discontinue the therapy but complete the follow-up assessments will be included in the intention-to-treat analysis.

#### Statistical analysis

First, student’s t-tests or appropriate non-parametric statistics will be used to evaluate the comparability of the intervention and control group at baseline in terms of sociodemographic and clinical characteristics. If, despite the stratified randomization procedure, the groups are not comparable on one or more background variables, those variables will be employed routinely as covariates in subsequent analyses.

Questionnaire scores will be calculated according to published scoring algorithms. Between-group differences over time in mean scores will be tested using multilevel analysis. Effect sizes will be calculated using standard statistical procedures. All analyses will, to as great an extent as possible, be conducted on an intention-to-treat basis. Per protocol analyses will also be carried out (as a secondary analysis), comparing women who meet minimal compliance levels with the program with the control group. We will use correlation analyses to examine the relationship between degree of program adherence, partner involvement, and program effect. For the analysis of the secondary outcome measures, appropriate statistical (p value) adjustments will be made for multiple testing. The semi-structured interview data will be transcribed and content analyzed to extract narrative, qualitative information about the women’s experience with the intervention.

## Discussion

A relatively large percentage of breast cancer survivors experience sexual problems as a consequence of their disease and its treatment. Studies show that CBT is an effective treatment method for alleviating sexual dysfunctions in the general population, when provided in a face-to-face setting. Recently, more attention has been paid to developing internet-based interventions targeting sexual functioning. However, research into internet-based interventions for FSDs is scarce, and even less is known about the efficacy of internet-based CBT specifically targeted at breast cancer survivors. In the current trial, we are investigating the efficacy of an internet-based CBT in reducing problems with sexuality and intimacy, psychological distress and menopausal symptoms, and in improving body image, marital functioning and health-related quality of life of breast cancer survivors.

This trial has several notable strengths, including: (1) the randomized trial design, (2) the multicenter nature of the trial, (3) the comparison of the intervention group with a waiting-list control group, (4) the use of intention-to-treat analyses, and (5) the long-term follow-up assessments of outcomes in women in the intervention group.

This trial also has several limitations. First, it would be valuable to compare the internet-based CBT group not only with a control group, but also with a face-to-face CBT group. However, our previous experience in offering breast cancer survivors the opportunity to participate in face-to-face sexual therapy proved problematic [de Blok G. Thesis on the outpatient clinic for sexuality and breast cancer of The Netherlands Cancer Institute. Unpublished manuscript]. Very few women were willing to take that step, indicating that they found the face-to-face setting too confronting. Thus, we anticipated that including a face-to-face therapy arm in the trial would result in substantial recruitment problems. Also, we consider it important to first establish the efficacy of the internet-based CBT program. If the program proves to be efficacious, a subsequent step could be a comparative effectiveness study with face-to-face treatment [61]. Second, although as one of the conditions for participating in the trial, women are asked not to participate in any other programs targeted at their sexual problems, the possibility exists that some women (particularly in the control group) may do so. However, we do not expect any such activities to be as structured, tailored and targeted at sexual problems specifically after breast cancer treatment as our CBT program. In any case, at each assessment point, women are asked to report any activities that they may have undertaken to alleviate their sexual problems. Third, the absence of T3 and T4 follow-up assessments for the control group precludes a longer-term between-group comparison of study outcomes. As noted earlier, this decision was based on both ethical and feasibility considerations. We (and the institutional review board) did not consider it appropriate to withhold therapy for an extended period of time, which would have been the case if women in the control group were required to complete all assessment points before having the opportunity to participate in the internet-based CBT program. We also believed that such a long waiting list period would have a significant, negative effect on recruitment into the study.

In conclusion, given the high rates of sexual dysfunction in breast cancer survivors, there is a need for effective and accessible treatments for these problems. If proven to be effective, internet-based CBT can be a valuable addition to the standard care offered to breast cancer survivors. Hopefully, this treatment will lower the barrier to seeking help, resulting in an improved quality of life after treatment of breast cancer.
